# TUBA1C: a new potential target of LncRNA EGFR-AS1 promotes gastric cancer progression

**DOI:** 10.1186/s12885-023-10707-7

**Published:** 2023-03-20

**Authors:** Haodong Wang, Huaiping Cui, Xinjun Yang, Lipan Peng

**Affiliations:** Shandong Provincial Hospital Affiliated to Shandong First Medical University, 250000 Jinan, Jinan China

**Keywords:** Gastric cancer, LncRNA EGFR-AS1, TUBA1C, Cell cycle

## Abstract

**Background:**

The lack of obvious symptoms of early gastric cancer (GC) as well as the absence of sensitive and specific biomarkers results in poor clinical outcomes. Tubulin is currently emerging as important regulators of the microtubule cytoskeleton and thus have a strong potential to be implicated in a number of disorders, however, its mechanism of action in gastric cancer is still unclear. Tubulin alpha-1 C (TUBA1C) is a subtype of α-tubulin, high TUBA1C expression has been shown to be closely related to a poor prognosis in various cancers, this study, for the first time, revealed the mechanism of TUBA1C promotes malignant progression of gastric cancer in vitro and in vivo.

**Methods:**

The expression of lncRNA EGFR-AS1 was detected in human GC cell lines by qRT–PCR. Mass spectrometry experiments following RNA pulldown assays found that EGFR-AS1 directly binds to TUBA1C, the CCK8, EdU, transwell, wound-healing, cell cycle assays and animal experiments were conducted to investigate the function of TUBA1C in GC. Combined with bioinformatics analyses, reveal interaction between Ki-67, E2F1, PCNA and TUBA1C by western blot. Rescue experiments furtherly demonstrated the relationship of EGFR-AS1and TUBA1C.

**Results:**

TUBA1C was proved to be a direct target of EGFR-AS1, and TUBA1C promotes gastric cancer proliferation, migration and invasion by accelerating the progression of the cell cycle from the G1 phase to the S phase and activating the expression of oncogenes: Ki-67, E2F1 and PCNA.

**Conclusion:**

TUBA1C is a new potential target of LncRNA EGFR-AS1 promotes gastric cancer progression and could be a novel biomarker and therapeutic target for GC.

**Supplementary Information:**

The online version contains supplementary material available at 10.1186/s12885-023-10707-7.

## Background

According to the 2020 global cancer statistics, gastric cancer (GC) is the fourth most commonly diagnosed malignant neoplasm (5.6%) and the fourth leading cause of cancer deaths (7.7%) [1]. The poor prognosis of GC is primarily attributable to cellular phenotypes such as aggressive proliferation and infiltration [2]. As a result of the inconspicuous early symptoms of GC and the lack of sensitive early diagnosis and detection methods, most GC patients are diagnosed at an advanced stage and miss the optimal treatment window [3–5]. Therefore, it is crucial to elucidate the molecular mechanisms underlying GC tumorigenesis and progression to develop novel biomarkers for early diagnosis and treatment.

LncRNAs are exceeding 200 nucleotides in length and do not acquire the ability to encode proteins. LncRNA has been described to be participated in various physiological and pathological processes at the cellular level through adjusting the expression of target genes at the transcriptional, after transcriptional and translational levels by interacting with DNA, miRNA or proteins [6]. LncRNA EGFR-AS1 derived from the reverse strand of lncRNA EGFR [7], it has been reported to regulate the expression of EGFR via heightening EGFR mRNA stability to active phosphatidylinositol-3 kinase (PI3K)/AKT pathway for the furtherance of the malignant progression of GC [8].

Microtubules are cytoskeletal filaments with an outer diameter of approximately 25 nm and are composed of heterodimers of globular α- tubulin and β- tubulin molecules[9]. Microtubules carry out a wide variety of key functions in virtually every cell and are essential for cell polarity, cell shape, and intracellular transport [10]. They are involved in the assembly of essential intracellular structures, such as mitotic and meiotic spindles, which play a key role in ensuring that cells divide correctly [11],axonemes, which are the central molecular machines of cilia and flagella [12],centrioles, which are the core structures of centrosomes.TUBA1C, a subtype of α-tubulin, is a special type of tubulin that has been extensively studied in association with several types of cancer. Recent studies have demonstrated that TUBA1C is an important mediator of cell cycle signaling, and it is aberrantly expressed in several malignancies, such as lung adenocarcinoma (LUAD), mammary cancer, and low-grade glioma and is also involved in the growth, migration, and invasion of tumor cells [13–15]. High TUBA1C expression has also been reported in hepatocellular carcinoma (HCC) and pancreatic ductal adenocarcinoma (PDAC), where it was an indicator of the poor prognosis and facilitated tumor cell proliferation and migration [16, 17]. However, there is no literature reporting the role of TUBA1C molecule in the development and metastasis of gastric cancer. In the present study, we discovered that LncRNA EGFR-AS1 directly binds to TUBA1C and affects its expression, in addition, TUBA1C-expression is significantly increased in GC cells and potentially promotes the proliferation, infiltration, and metastasis of GC cells. The results obtained in this study are consistent with the TCGA database. The matescape database was used to investigate the potential role of TUBA1C in GC[18]. Further analysis in vitro showed that TUBA1C influenced the expression of the oncogenes: Ki-67, E2F1 and PCNA, and promote malignant progression of gastric cancer cells by affecting the cell cycle. The E2F transcription factors (E2Fs), which have been reported to bind to the promoter region in adenoviruses [19], are a class of transcription factors that are essential for cellular multiplication, differentiation, and apoptosis. E2F1 is a type of E2F that plays a role in the regulating of periodic expression of genes required for cell proliferation [20]. The E2F1 has been reported as a target for the retinoblastoma (Rb) pocket protein [21, 22], which is essential for cell cycle regulation and a key mutant tumor suppressor in several malignancies [23]. The transcription factor E2F1 is suppressed on interacting with Rb protein. The PCNA and Ki-67 genes have been suggested as molecular markers for cell proliferation [24, 25]. Finally, tumor xenograft models demonstrated that reduced levels of TUBA1C inhibited tumor growth in vivo. Collectively, our experimental results illustrate that TUBA1C may potentially serve as a novel biomarker for early diagnosis and as a new therapeutic target for GC.

## Materials and methods

### Human GC samples

Thirty pairs of tumors and corresponding adjacent nontumorous tissues were collected from GC patients at Shandong Provincial Hospital from 2019 to 2021. All patients had not received chemotherapy treatment before surgery. All of the collected tissue samples were immediately preserved in liquid nitrogen and conserved in -80 °C fridge. The study has been ratified by the Research Ethics Review Board of Human Subjects at the Shandong Provincial Hospital, and all of the participants signed a written consent form.

### Cell culture

Gastric cancer cell lines (HGC-27, BGC-823, MGC-803, MKN-45, and SGC-7901) and GES-1 cell line were purchased from the Institute of Cells, Chinese Academy of Sciences (Shanghai, China). ALL cells were cultivated in RPMI-1640 medium (Gibco, CA, USA) supplementary with 10% fetal bovine serum, 100 mg/mL streptomycin and 100 U/mL penicillin (Gibco, CA, USA) in 5% CO2 at 37 ° C[26].

### RNA extraction and qPCR

The total RNA was extracted from fresh GC tissues and cell lines using the Trizol reagent (Takara, Japan). Reverse transcription was performed to obtain cDNA by using the Evo M-MLV RT Premix (AG, China).QRT-PCR was carried out using SYBR Green Pro Taq HS Premix (AG, China) with an LightCycler 480 system (RD, Basel, Switzerland). The primer sequences used for qRT-PCR were listed in supplementary Table 1.

### Cell transfection

Small interfering RNA anti EGFR-AS1 (si-EGFR-AS1) and TUBA1C (si-TUBA1C) as well as negative control RNA (si-NC) and EGFR-AS1 overexpression plasmid(oe-EGFR-AS1) and TUBA1C overexpression plasmid (oe-TUBA1C) were designed and synthesized by Genomedtech (Shanghai, China). Cell transfection was conducted when the cell density achieved 80%. Transfection efficiency was verified using western blotting or qRT-PCR. All interfering sequences plasmid sequences were shown in supplementary Table 2.

### RNA pull-down and mass-spectrometry assay

The plasmid pcDNA3.0 containing sense or antisense sequence was designed and synthesized by Genomeditech (Shanghai, China). The RNA sequences were linearized with the FastDigest XhoI Kit (ThermoFisher Scientific, Waltham, MA, USA) and T7 MEGAscript Kit (Invitrogen, USA), and purified with Purification Kit (TianGen Biotech, Beijing, China). The RNA pull-down assay was accomplished by using the Magnetic RNA-Protein Pulldown Kit (Thermo Scientific, USA) based on the manufacturer’s instructions. Briefly, biotinylated RNA was captured on streptavidin magnetic beads and was then incubated with cell lysates at 4 °C for 6 h before washing and elution of RNA–protein complexes. The proteins interacting with lncRNAs were detected and analyzed by mass spectrometry at Shandong University(Shandong, China). Briefly, Proteins were digested with trypsine(Promega). Peptides were desalted and concentrated using C18-based solid phase extraction prior to analysis by high resolution/high mass accuracy reversed phase (C18) nano-LC-MS/MS. Peptides were separated on a C18 column at 250 nl/min with a gradient increasing from5% Buffer B/95% buffer A to 35% buffer B/65% Buffer A in typically 120 min (buffer A: 0.1% formic acid in water, buffer B: 0.1% formic acid in acetonitrile).Mass spectrometers (Orbitrap elite, Thermo Scientific) were operated in data dependent (DDA) positive ion mode. All raw fifiles were processed using Proteindiscover (version 1.4, Thermo-Scientific) for database searching. MS/MS spectra were searched against the UniProtKB/Swiss-Prot human database (down loaded in December 29, 2017). The extracted protein was separated on polyacrylamide gel electrophoresis (PAGE) gels and stained with a silver staining kit (Thermo Scientific, USA). The proteins identified were listed in supplementary Table 3.

### Western blot

Whole protein was extracted from transfected cells after cells were lysed using the RIPA buffer containing PMSF and phosphatase inhibitors. After quantifying by the BCA Protein Assay Kit (Beyotime, Shanghai, China), the proteins were resolved by using 10% sodium dodecyl SDS-PAGE, and then proteins were transferred to PVDF membranes. For clearer western blot bands, the blots were cut prior to hybridisation with antibodies. Membranes were incubated with specific primary antibodies ( anti-TUBA1C (Proteintech, Wuhan, China), anti-E2F1 (Proteintech), anti-ki-67 (Proteintech), anti-PCNA (Proteintech), GAPDH (Proteintech), and β-actin (Proteintech)) diluted in antibody diluent at 4 °C for 12 h. Then incubating the membranes with a secondary antibody for 2 h. The western blot bands were scanned by Amersham Imager 600 and analyzed by Image J software.

### Immunohistochemistry (IHC) staining

Immunohistochemistry was performed using an immunohistochemical kit (Zsgb Bio, Beijing, China). In brief, the slices are incubated in sequence with primary antibodies and secondary antibodies,then the slices were stained by DAB (SP kit, ZSGB-BIO, China) as a chromogen and washing the coloring board with water, subsequently ,the slices were soaked in hematoxylin for staining. Finally,dehydrated and sealed with a coverslip.The immunostaining score was evaluated by two independent pathologists from Provincial Hospital affiliated to Shandong First Medical University.

### CCK-8 assay

The degree of cell growth was monitored using the cell counting kit-8(CCK-8) (Kumanoto, Japan). The transfected cells were inoculated into 96-well plates (3,000 cells/well), and cultured for 24, 48, 72, 96 h, subsequently, 10 µl CCK-8 was added and incubated for 1 h in the dark at 37 °C. Following incubation, cell proliferative viability was estimated by measuring the absorbance at 450 nm.

### EDU cell proliferation

The EDU Cell Proliferation Assay kit (Invitrogen, USA) was used to analyze cell multiplication. Following transfection 48 h, cells were seeded into 96-well plates at a concentration of 5,000 cells per well.and continue to cultivate for 24 h,after this, 2 h incubation with 50 µM EDU in 5% CO2 at 37 ° C and the cells are washed thrice with PBS, fixed using 4% polyacetaldehyde for 30 min, then incubated with 0.5% Triton-X-100 PBS for 20 min, finally stained with Apollo Dye Solution. Results were observed using high-content imaging microscopy.

### Transwell assay

Transwell chambers with 8 μm aperture (Transwell, Costar, UK) were used to estimate the migratory and invasive ability of the cells. For the invasion, Matrigel (3 mg/ml) (BD, Nanjing, USA) was added to the upper chamber at 37 °C for 1 h and GC-transfected cells or control cells (1 × 10^5^) were placed in the upper chamber. Following 24 h of incubation, cells in the chamber were fixed using 4% paraformaldehyde for 30 min and stained using crystal violet for 30 min. For the migration assays, GC-transfected cells or control cells (4 × 10^4^) placed in the upper chambers were suspended in a 200 µl serum-free medium ,10% fetal bovine serum was added as a chemotactic agent to the lower compartment of the medium. Following 24 h of incubation, cells in the upper cavity were scraped with a cotton swab, fixed using formaldehyde fixation for 30 min and crystalline purple was added for 30 min. Photographs was performed under the microscope (Olympica, Tokyo, Japan) using 200× magnification.

### Scratch healing assay

Scratch healing assay was performed to estimate the cell migration capacity. When transfected gastric cancer cells and their control cells were grown to 90% confluency in 6-well plates, make a straight scratch in wells by using a sterilized 200 µl pipette tip. After washing floating cells with PBS, incubated at 37 °C, 5% CO2for 24 h. Photographs were taken by a digital microscopy at 0 and 24 h.

### Cell cycle

Cell cycle was analyzed using the Cell Cycle Detection kit (Keygen Biotech, Nanjing, China). Briefly, treated cells were harvested and adjust the cell concentration to 1 × 10^6^ cells/ml. Then, fixed the cells overnight at 4 °C in 70% cold ethanol. Fixed cells were washed twice in PBS, treated with PI/RNase A staining solution for 1 h and immediately analyzed by flow cytometry.

### Tumorigenesis and metastasis in vivo

Validated by animal experiments at Shandong First Medical University. Briefly, MKN-45 cells transfected with si-ctrl or si-TUBA1C were pooled and suspended in PBS on ice.5 × 10^6^ cells were injected subcutaneously into the bilateral armpits of six mouse. Tumor volume was monitored and recorded weekly after injection. Mice were sacrificed by CO 2 inhalation followed by cervical dislocation after 4 weeks and tumors were removed for further western blotting and IHC These studies were conducted according to the ethical principles of the Declaration of Helsinki also approved by the Ethics Review Committee of the Provincial Hospital affiliated to Shandong First Medical University.

### Statistical analysis

All data were analyzed in SPSS 26.0 (SPSS, Chicago, Illinois, USA). Students t-test was used to measure the statistical significance between groups. All the experiments were repeated at least three times,and all results were presented as mean values ± standard deviation of at least three independent experiments.P-values < 0.05 were recognized as significant (*p < 0.05, **p < 0.01 and ***p < 0.001).

## Results

### LncRNA EGFR-AS1 is upregulated in gastric cancer tissues

**Fig. 1:**
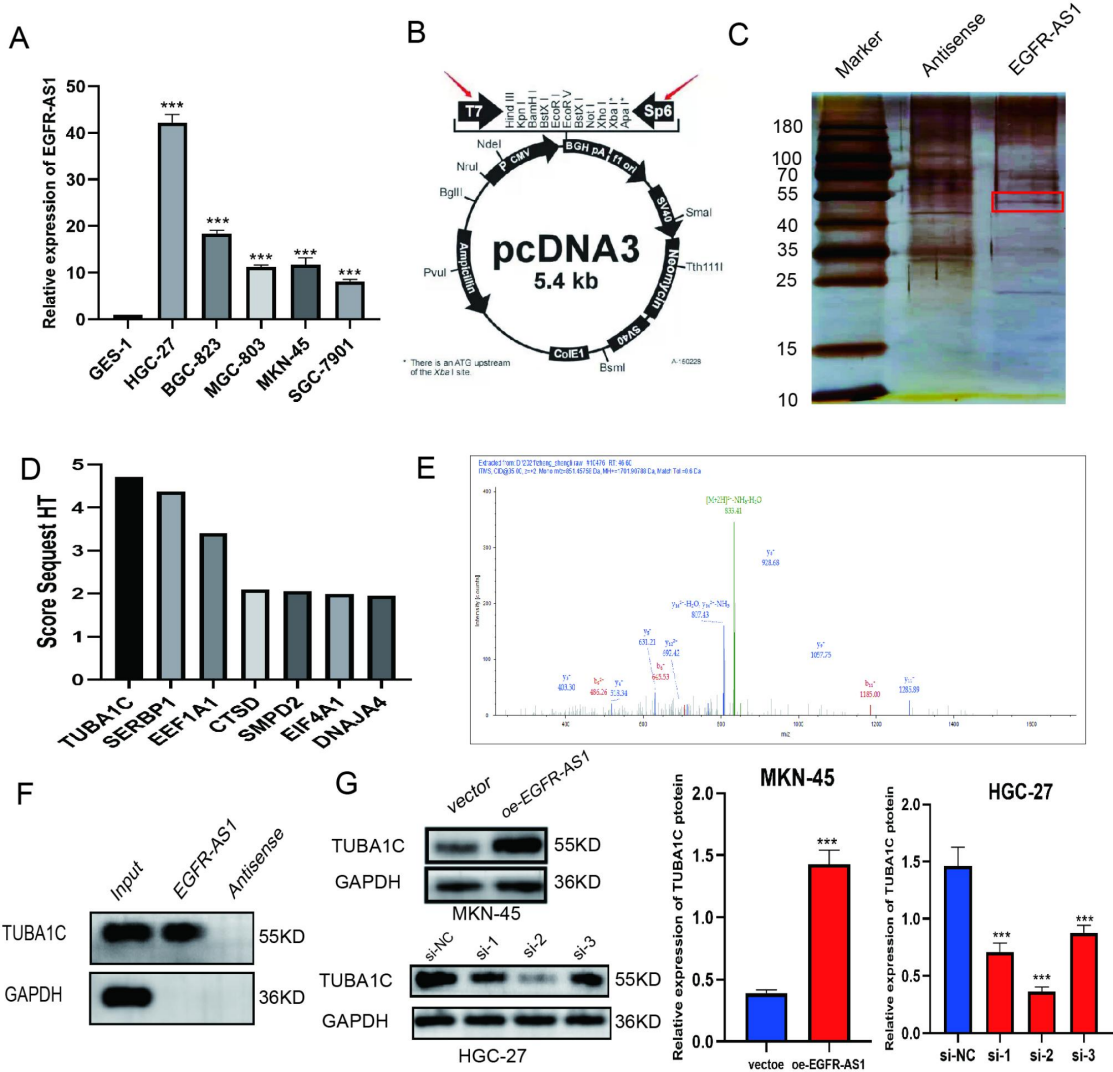
**LncRNA EGFR-AS1 binds to TUBA1C protein. A** LncRNA EGFR-AS1 expression was detected by qRT-PCR in GC cell lines(HGC-27, BGC-823, MGC-803, MKN-45 and SGC-7901) and the normal gastric cell line (GES-1). **B** Schematic representation of the plasmid contained EGFR-AS1 or its antisense sequence. **C** The expression of the protein obtained by the pull-down method in HGC-27 cells was detected by silver staining. **D** Top 10 reliable proteins identified by mass spectrometry **E** TUBA1C was identified as A EGFR-AS1-binding protein. **F** RNA-pulldown followed by Western blot analysis to identify the EGFR-AS1–TUBA1C interaction **G** The effects of EGFR-AS1 overexpression and knockdown on TUBA1C protein expression. (The blots were cut prior to hybridisation with antibodies.) (oe = overexpression, NC = negative control).Data are mean ± SD. (Student’s t test, ***p < 0.001)

Formerly, lncRNA EGFR-AS1 was shown to have a definite facilitated function in both the cytoplasm and nucleus of GC cells. EGFR-AS1 induced cell proliferation by activating the EGFR/PI3K/AKT pathways in vitro as well as in vivo. Subsequently, a significantly increased expression of EGFR-AS1 in human GC cell lines compared to GSE-1 and was confirmed using qPCR (Fig. 1A).

### LncRNA EGFR-AS1 directly binds to TUBA1C protein

LncRNAs have been reported to regulate protein stability by binding to tumor-associated proteins directly [27]. In this study, the RNA pulldown assay was used to identify the protein that binds to EGFR-AS1. The experimental cell line was the HGC-27 cell line owing to a high EFGR-as1 expression. First, an exogenous plasmid containing the EGFR-AS1 or its antisense sequence was synthesized (Fig. 1B). Finally, the biotin-labeled EGFR-AS1 and intron control were incubated with HGC-27 cell lysates, and the enriched products were identified using 10% SDS-PAGE electrophoresis and silver staining. (Fig. 1C). Results from the mass spectrometry (MS) analysis indicated the presence of several proteins, and we picked proteins that were strongly associated with cancer in differential bands,including TUBA1C, SERBP1, EEF1A1, CTSD, SMPD2, EIF4A1, and DNAJA4, out of which TUBA1C was the most abundant (Fig. 1D ) and the mass spectrum is obtained by separation according to the mass-to-charge ratio .(Fig. 1E). To validate our results, we performed a second independent RNA pulldown assay and WB analysis to verify the EGFR-AS1-TUBA1C interaction(Fig. 1F). For follow-up experiments, TUBA1C.

expression in the EGFR-AS1-upregulated MKN-45 cell line and the EGFR-AS1-downregulated HGC-27 cell line was measured by using WB ( Fig. 1G ).

### TUBA1C is upregulated in gastric cancer tissues

**Fig. 2:**
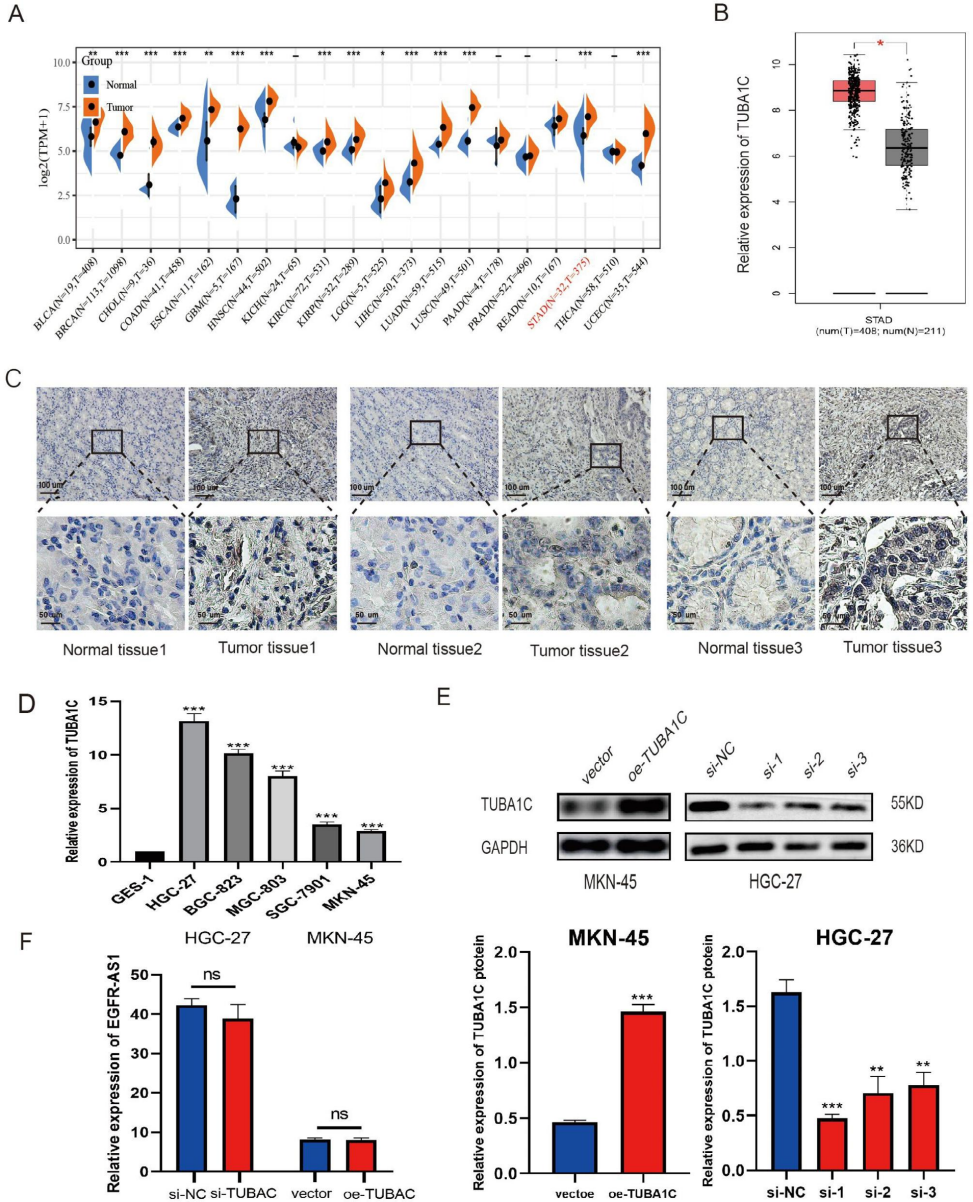
**TUBA1C is upregulated in GC. A, B** Relative expression of TUBA1C in multifarious human tumors from TCGA database. **C** IHC staining of TUBA1C in paired normal tissues and GC tissues. **D** TUBA1C expression was detected by qRT-PCR in GC cell lines(HGC-27, BGC-823, MGC-803, SGC-7901 and MKN-45) compared with GES-1.**E** TUBA1C overexpression and knockdown efficiencies of transduced cells were confirmed by Western blotting. (The blots were cut prior to hybridisation with antibodies.) **F** TUBA1C does not affect the expression of EGFR-AS1 in gastric cancer.Data are mean ± SD. (Student’s t test, ns means no significant difference,**p < 0. 0 1, ***p < 0.001)

Bioinformatic analysis was performed to determine the function of TUBA1C in GC patients. TCGA databases also show that TUBA1C mRNA is upregulated in a variety of malignancies including colon, esophagus, lung, hepatocellular and gastric cancers. (Fig. 2A),and we affirmed that TUBA1C was highly expressed in GC tissues compared to gastric normal tissues by using GEPIA tool(Fig. 2B). Furthermore, immunohistochemistry (IHC) showed the expression of TUBA1C in human GC tissues was significantly higher than human gastric normal tissues (Fig. 2C). Moreover, the qRT-PCR analysis showed that TUBA1C expression is significantly higher in GC cell lines (HGC-27, BGC-823, MGC-803, SGC-7901, and MKN-45) than in normal gastric epithelial cell lines (Fig. 2D). Among the GC cell lines, HGC-27 showed the highest TUBA1C expression level, and MKN-45 cells showed the lowest TUBA1C expression level, therefore, these two cell lines were selected for further analysis.

Recent studies have revealed that TUBA1C is involved in the cell cycle and cell proliferation in a variety of cancers and its upregulation can have dramatic effects on tumor growth and progression [28]. Furthermore, TUBA1C was found to be a key mediator in the cell cycle-signaling pathways, which show aberrant expression in multifarious malignant tumors; tumor multiplication, migration, and invasion; and as a novel target for tumor-targeted therapy. A previous study reported that changes in the expression of tubulin-isoform and microtubule dynamics are related to alterations in p53 [29]. The function of TUBA1C in the tumorigenesis of solid tumors such as liver, breast, ovarian, and lung adenocarcinomas have been described previously; however, its role in GC remains to be explored. In order to assess the function of TUBA1C in GC metastasis, an optimal siRNA, based on the TUBA1C knockdown efficiency, was selected for in vitro experiments. TUBA1C expression in the TUBA1C-upregulated MKN-45 cell line and the TUBA1C-downregulated HGC-27 cell line was measured using WB (Fig. 2E). The qRT-PCR results showed that the overexpression and knockdown of TUBA1C had no significant effect on the mRNA expression of EGFR-AS1 in HGC-27 and MKN-45 cells (Fig. 2F).

### TUBA1C promotes GC cells proliferation, migration, and invasion in vitro

**Fig. 3:**
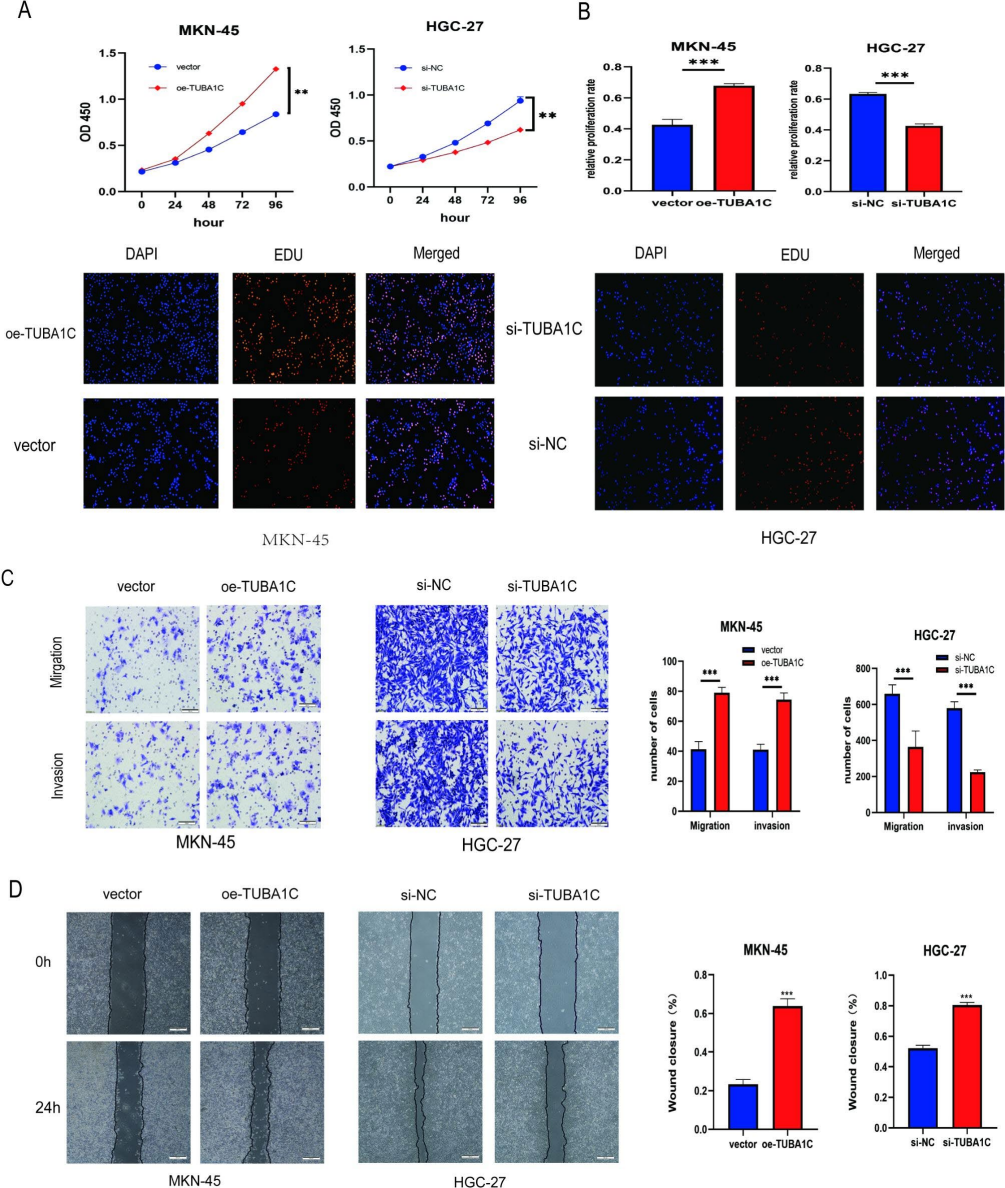
**TUBA1C promotes proliferation, migration, and invasion of GC cells in vitro. A** CCK-8 assays were performed to test cell proliferation of MKN-45 cells and HGC-27 cells after transfection with different vectors. **B** EUD assays was used to detect DNA synthesis of MKN-45 cells and HGC-27 cells after transfection with different vectors. **C, D** Transwell assays and scratch wound healing assays to detect the invasion and migration abilities of the MKN-45 cells and HGC-27 cells after treatment. Data are mean ± SD. (Student’s t test, **P < 0.01, ***P < 0.001 )

Experiments with CCK8 and EDU showed that TUBA1C knockdown inhibited proliferation in the HGC-27 cell line, while TUBA1C overexpression significantly promoted cell proliferation (Fig. 3A and B). Transwell experiments showed that TUBA1C knockdown significantly inhibited HGC-27 cell migration and infiltration, while overexpression significantly promoted cell migration and infiltration (Fig. 3C). Wound-healing experiments showed that a decrease in TUBA1C inhibited HGC-27 cell migration, while its overexpression significantly accelerated cell migration in the MKN-45 cell line (Fig. 3D). In conclusion, this data indicate that the regulation of TUBA1C contributes to GC progression.

### TUBA1C regulated cell cycle progression and promoted the expression of E2F1,Ki -67 and PCNA

**Fig. 4:**
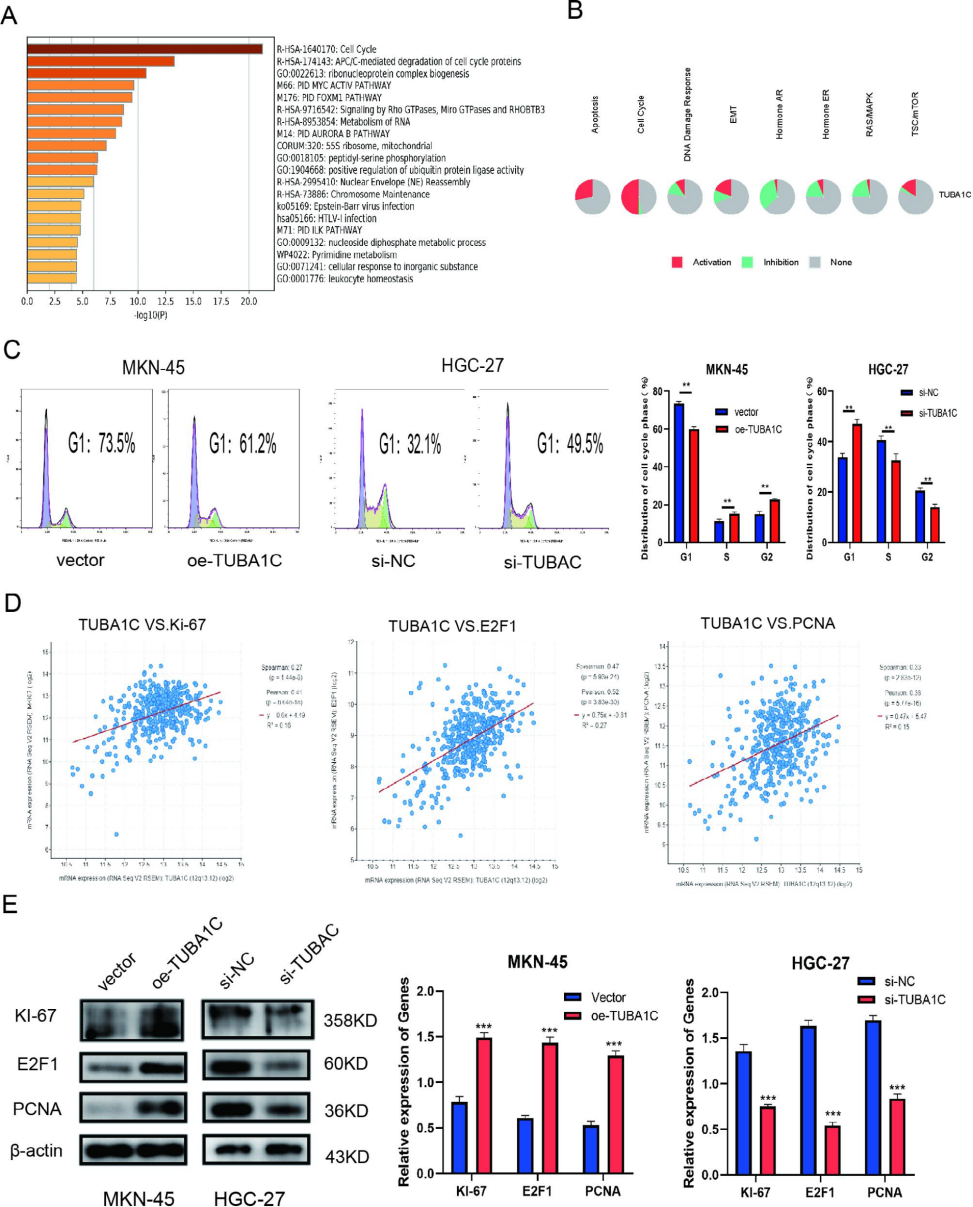
**Bioinformatics prediction and experimental verification of TUBA1C. A** Functional pathway analysis of TUBA1C was performed using matescape enrichment analysis. **B** Pathway activity of the TUBA from GSCA. **C** Cell cycle was determined in MKN-45 and HGC-27 cells transfected **D** Co-expression of TUBA1C with KI67, E2F1 and PCNA in GC were analyzed in cBioPortal using the TCGA database. **E** The expression of KI-67, E2F1 and PCNA in MKN-45 cells transfected with oe-TUBA1C and its control and HGC-27 cells transfected with si-TUBA1C and its control were detected by Western blotting. (The blots were cut prior to hybridisation with antibodies.) Data are mean ± SD. (Student’s t test **P < 0.01, ***P < 0.001 )

Biological information analysis were utilized to explore the downstream mechanism of TUBA1C.The preliminary results was performed using matescape database ( http://www.metascape.org/ ) show the “cell cycle process” pathway as enriched with an increase in TUBA1C(Fig. 4A), and pathway activity from GSCA (Fig. 4B)also shows that TUBA1C is highly correlated with the cell cycle. Thus, we further investigated the role of TUBA1C in regulating cell cycle progression of GC cells. We found that knockdown TUBA1C causes HGC-27 cells to arrest in G1 phase while overexpressing TUBA1C accelerates the progression of the cell cycle from the G1 phase to the S phase resulting in cell proliferation(Fig. 4C). TUBA1C was found to be potentially associated with the dysregulation of well-known cancer genes such as Ki-67,E2F1 and PCNA by using the cBioPortal ( https://www.cbioportal.org/ )(Fig. 4D). E2F is the first cell protein combined with PRB tumor-inhibitory factors. E2F1 is a type of E2F, which is known to adjust the target gene through a variety of signaling pathways, for instance, cell cycle, apoptosis, and differentiation [30]. Because E2F1 has tumor stimuli, its expression participates in preventing of proliferation and apoptosis of tumor cells [31]. On the other hand, Ki-67and PCNA have been extensively used as markers of proliferation[32]. Through WB experiment, the present study showed that the knockdown of TUBA1C in HGC-27 cells significantly reduced the expression of Ki-67, E2F1 and PCNA proteins, on the contrary, the expression of Ki-67,E2F1and PCNA in TUBA1C-overexpressed MKN-45 cells is increased (Fig. 4E). Analysis of the TCGA database yields similar results. In conclusion, TUBA1C promotes GC by accelerating the progression of the cell cycle from the G1 phase to the S phase and activating the expression of Ki-67, E2F1, and PCNA.

### LncRNA EGFR-AS1 promotes GC cells proliferation, invasion, and metastasis as well as Ki-67, E2F1 and PCNA expression by targeting TUBA1C

**Fig. 5:**
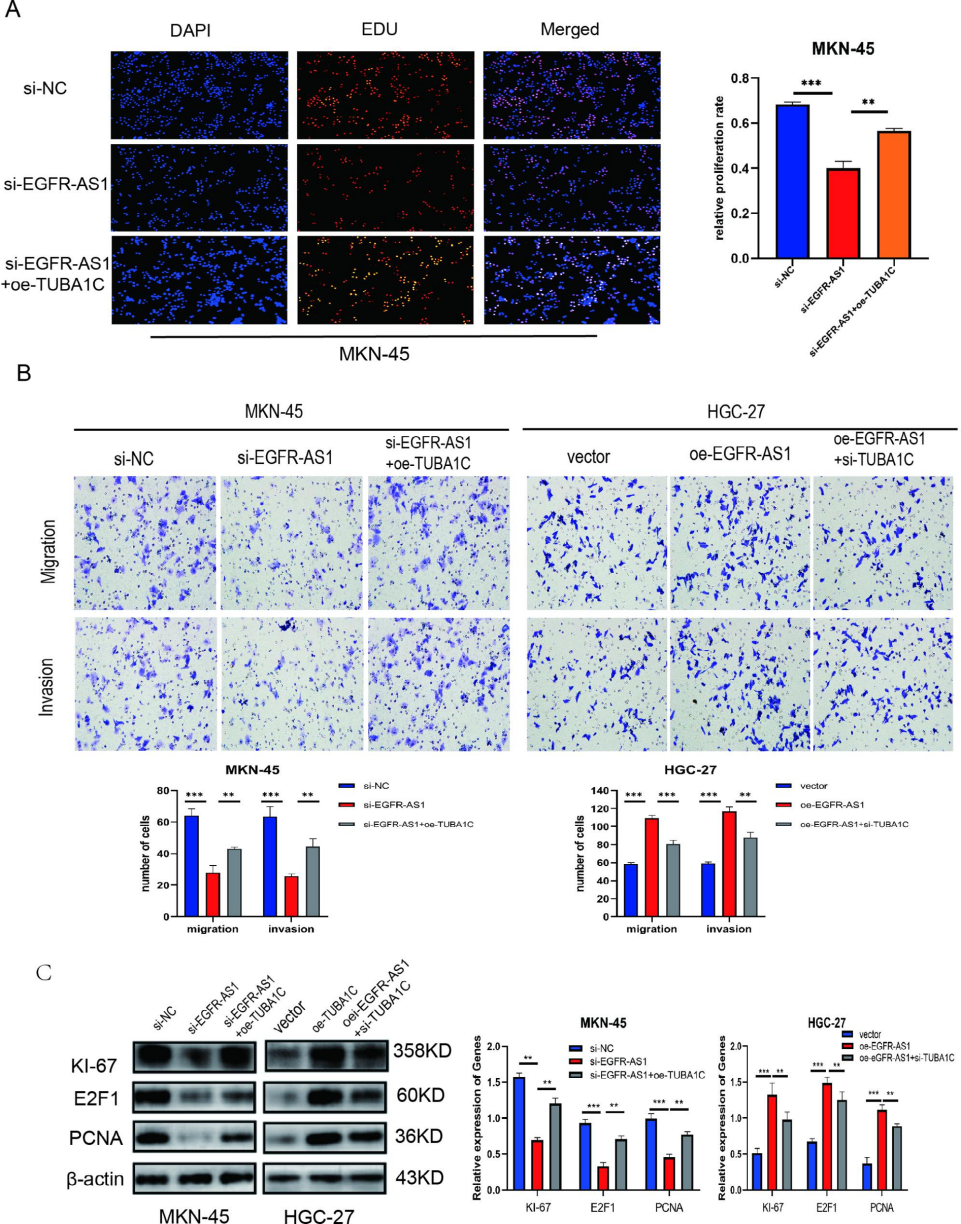
**LncRNA EGFR-AS1 facilitates the proliferation, infiltration, and migration of GC cells and promotes the expression of Ki-67**, **E2F1and PCNA by targeting TUBA1C. A** EDU rescue assay was performed to detect proliferation of si-EGFR-AS1,si-NC and si-EGFR-AS1 + oe-TUBA1C-transfected MKN-45 cells. **B** Migration and invasion of si-NC, si-EGFR-AS1 or si-EGFR-AS1 + oe-TUBA1C transfected MKN-45 cells and vector,oe-EGFR-AS1,or oe-EGFR-AS1 + si-TUBA1C transfected HGC-27 cells was detected by transwell rescue assay. **C** The expression of KI-67, E2F1and PCNA in MKN-45 cells transfected with si-NC, si-EGFR-AS1 or si-EGFR-AS1 + oe-TUBA1C and HGC-27 cells transfected with vector,oe-EGFR-AS1,or oe-EGFR-AS1 + si-TUBA1C were detected by Western blotting.(The blots were cut prior to hybridisation with antibodies.) Data are mean ± SD. (Student’s t test **P < 0.01, ***P < 0.001 )

The rescue experiment was designed to test whether EGFR-AS1 could target TUBA1C and promote proliferation, infiltration, and metastasis in GC cells. In EDU experiments, overexpressed-TUBA1C reversed the growth-promoting effect of EGFR-AS1-downregulated MKN-45 cells (Fig. 5A). In transwell experiments, overexpressed-TUBA1C reversed the infiltration and metastasis-promoting effect in EGFR-AS1-downregulated MKN-45 cells, whereas the promotional effect induced by the upregulation of EGFR-AS1 in HGC-27 cells was partially offset by knockdown of TUBA1C (Fig. 5B). The above experiments show that TUBA1C promotes the expression of Ki-67, E2F1 and PCNA. The effect of TUBA1C knockdown or overexpression on Ki-67, E2F1 and PCNA expression was also found to be reversed by EGFR-AS1 overexpression and silencing (Fig. 5C). Taken together, EGFR-AS1 facilitates the multiplication, infiltration, and migration of GC cells and promotes the expression of Ki-67, E2F1 and PCNA through TUBA1C-targeting.

### Knockdown of TUBA1C inhibits the growth of GC in vivo

**Fig. 6:**
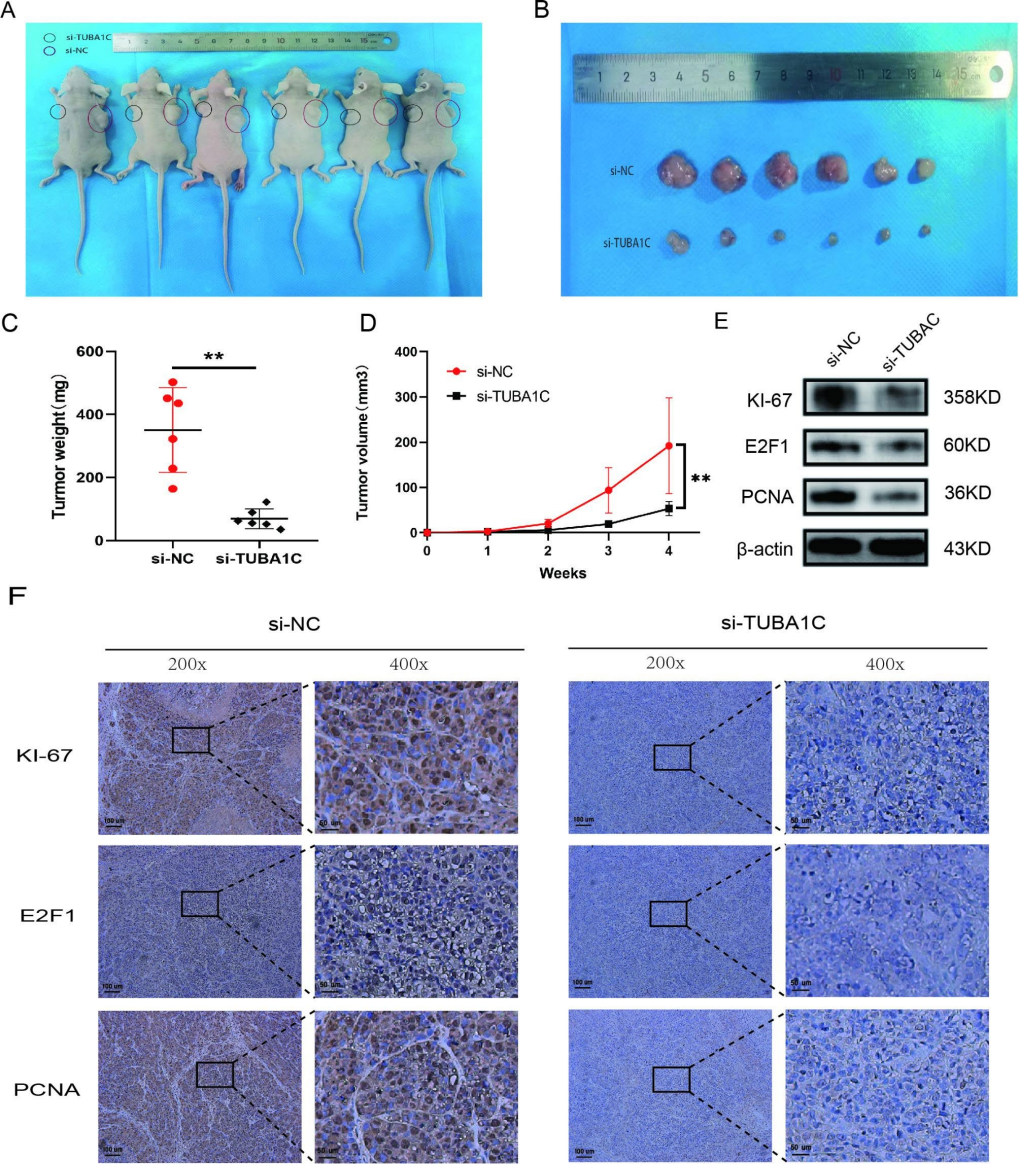
**TUBA1C knockdown significantly reduced growth of GC xenografts in nude mice. A, B** The mice were sacrificed after 4 weeks of subcutaneous injection of si-NC-transfected MKN-45 cells in the left armpit and subcutaneous injection of si-TUBA1C-transfected MKN-45 cells in the right armpit. **C, D** the weight of dissected tumors and the curve of tumor growth. **E, F** Expression of Ki-67, E2F1 and PCNA in mouse tumors was determined by WB and IHC.(The blots were cut prior to hybridisation with antibodies.)

A subcutaneous tumor model should be established and the role of TUBA1C in the body should be further elucidated. The TUBA1C-knockdown MKN-45 cells and negative control cells were inoculated in the nude rat to determine whether the TUBA1C knockout inhibits the proliferation of GC cells in the body. The tumor of the Si-TUBA1C group is significantly smaller than the control group (Fig. 6A-D). Furthermore, tumors from the control group showed higher Ki-67, E2F1 and PCNA expression level than tumors from the TUBA1C knockout group(Fig. 6EandF). These results confirm that TUBA1C contributes to malignant behaviours of GC cells by regulating the expression of multiple genes.

**Fig. 7:**
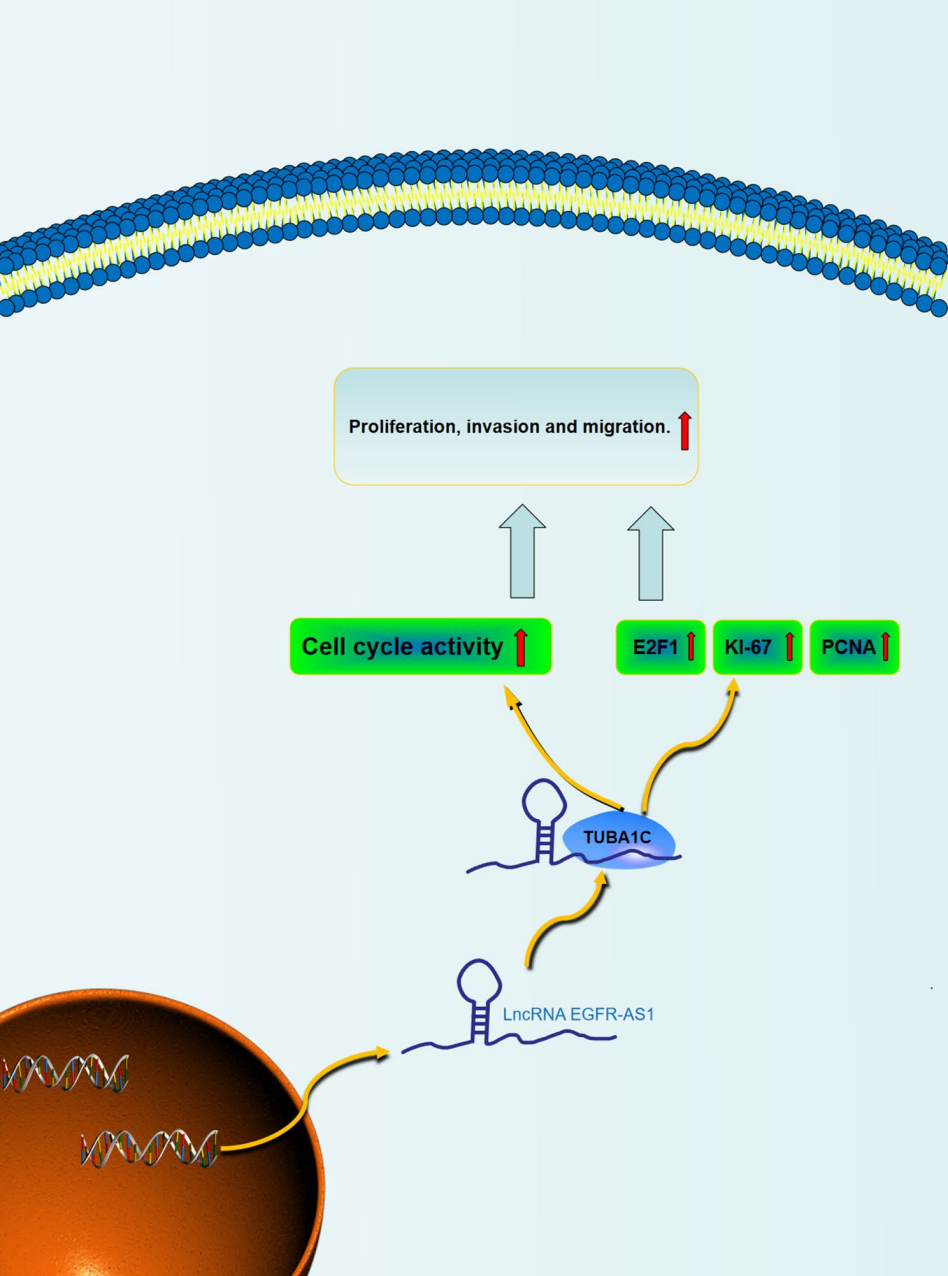
**Schematic diagram of EGFR-AS1-mediated GC progression.** EGFR-AS1 enhances the stability of EGFR mRNA to active PI3K/AKT pathway and binds to TUBA1C to promote the expression of E2F1, KI-67 and PCNA to promote the progression of GC.

## Discussion

The lack of obvious symptoms of early GC as well as the absence of sensitive and specific biomarkers that can predict prognosis and metastatic potential for gastric cancer diagnosis results in poor clinical outcomes. Consequently, it is crucial to elucidate the potential mechanism of malignant progression of GC. Recently, mounting evidence suggests that lncRNA played a pivotal role in tumorigenesis and progression of GC[33]. However, a great deal of lncRNAs that have been characterized and reported functional mechanisms have not been used to diagnose and treat gastric cancer patients, such as WFDC21P[34], UBE2CP3[35].Recently, Hu et al. confirmed that lncRNA EGFR-AS1 was obviously upregulated in GC and higher EGFR-AS1 expression was associated with poor prognosis[8]. Nevertheless, the mechanism of the high expression of EGFR-AS1 is unclear. On the other hand, the exact regulatory mechanism of EGFR-AS1 itself during GC progression has not been investigated.

Several studies have shown that a majority of the lncRNAs play a role in regulating the expression of target genes through trans-regulation, which predominantly occurs in association with specific proteins [36, 37]. Therefore, it is speculated that EGFR-AS1 potentially affects the expression of downstream genes by binding to proteins that subsequently promote GC progression. The RNA pulldown assay and mass-spectrometry analysis confirmed that out of the proteins that bind to EGFR-AS1, including TUBA1C, SERBP1, EEF1A1, CTSD, SMPD2, EIF4A1, and DNAJA4, several occur as lncRNA/protein complexes, and the TUBA1C-containing complex is the most common. This proteomic validation was used to select cancer-associated proteins and identified as the TUBA1C-test proteins, which directly bind to EGFR-AS1 in independent RNA pulldown assays. Subsequently, WB analysis was performed to confirm whether the EGFR-AS1 knockdown inhibited TUBA1C expression.

Tubulin is a major component of eukaryotic cells plays a key role in dynamic aggregation and disaggregation through influencing cell replication and division [38].TUBA1C is reported to be a multifunctional cytoskeletal protein and a α-tubulin isoform involved in the progression of mitosis and cell division [39]. High TUBA1C expression has been shown to be closely related to a poor prognosis in in various cancers, For example, the downregulation of TUBA1C inhibited the proliferation and migration of LUAD[13],hepatoma[17],and glioma[40] cells through cell cycle arrest, In addition, TUBA1C upregulates YAP expression and promotes aerobic glycolysis to promote breast cancer cell proliferation[14]. However, the role of TUBA1C in GC and its biological role in GC have not yet been elucidated. This study, for the first time, revealed that TUBA1C levels were elevated in GC tissues when compared with normal tissues. TUBA1C was also shown to promote the proliferation, invasion, and, migration of GC cells. Moreover, preliminary results from matescape enrichment analysis and co-expressed genes collected through the TCGA database, suggest that high expression of TUBA1C is enriched in the pathways associated with the “cell cycle process.“ We then validated the role of TUBA1C in the cell cycle and found that knockdown TUBA1C causes HGC-27 cells to arrest in G1 phase while overexpressing TUBA1C accelerates the progression of the cell cycle from the G1 phase to the S phase. These results are consistent with previous reports that proteins and protein kinases that regulate the cell cycle (cyclins D1 and E1 and CDKs 2, 4 and 6) expression were decreased following TUBA1C knockdown ,thereby affecting the cell cycle and inducing apoptosis[16]. Further analysis showed that TUBA1C could influence the expression of oncogene : Ki-67, E2F1 and PCNA. Meanwhile, TUBA1C expression may be correlated with P53 expression, since studies have reported complex changes in tubulin isoform expression and microstatic dynamics to be associated with changes in P53. Collectively, the above data suggest that TUBA1C is highly enriched in GC patients, TUBA1C promotes the migration and invasion and might induce the cell apoptosis by regulating cell cycle in GC cells. Then we verified the concept that EGFR-AS1 promote the proliferation, infiltration, and metastasis in GC cells through binding to TUBA1C by rescue experiments. Our findings may provide a basis for the development of more effective strategies for treating GC.

In spite of its promising findings, this study still has some limitations. The specific mechanism of EGFR-AS1 regulates TUBA1C has not been elucidated, besides, further studies are needed to investigate how EGFR-AS1 interacts with other proteins and how it is regulated by other transcription factors. Furthermore, the use of the MKN-45 cell line for animal studies may be controversial. Because we have found that the HGC-27 cell line is difficult to form tumors through experiments, and the mkn-45 cell line is widely used in animal experiments and is a mature cell line. In addition, all of our cell lines have higher expression levels than normal gastric cell line, which can also explain that mkn-45 can be knocked down from this perspective. So we finally decided to use mkn-45 instead of HGC-27, although not in line with conventional principles, can also show that the deletion of TUBA1C does affect the formation of tumors, and the conclusion is credible.

## Conclusion

We found TUBA1C is a new potential target of LncRNA EGFR-AS1 promotes gastric cancer progression, the cancer-promoting function of lncRNA EGFR-AS1 in GC is partially mediated by heightening EGFR mRNA stability to active PI3K/AKT pathway and EGFR-AS1-TUBA1C interactions. Our further experiments show that TUBA1C promotes gastric cancer by accelerating the progression of the cell cycle from the G1 phase to the S phase and activating the expression of oncogene: Ki-67, E2F1 and PCNA(Fig. 7), and this is the first report showing that TUBA1C is a novel GC marker for early diagnosis, prognosis and progression.

## Electronic supplementary material

Below is the link to the electronic supplementary material.

Supplementary Material 1

Supplementary Material 2

Supplementary Material 3

Supplementary Material 4

## Data Availability

The data used to support the findings of this study are included in the article. The datasets generated and analysed during the current study have been deposited to the ProteomeXchange Consortium via the PRIDE [41] partner repository with the dataset identifier PXD040199”.(Submission details: Project Name: TUBA1C: a new potential target of LncRNA EGFR-AS1 promotes gastric cancer progression Project accession: PXD040199 Project DOI: Not applicable Reviewer account details : Username:reviewer_pxd040199 @ebi.ac.uk Password: NtTZXRsR).

## Abbreviations

GC: gastric cancer
lncRNA: long noncoding RNA
LUAD: lung adenocarcinoma
PDAC: pancreatic ductal adenocarcinoma
si-RNA: Small interfering RNA
qRT-PCR: Quantitative real-time PCR
FBS: Fetal bovine serum
EdU: 5-Ethynyl-2′- deoxyuridine
CCK-8: Cell Counting Kit-8
IHC: Immunohistochemistry
