# Supplementary material for: TUBA1C: a new potential target of LncRNA EGFR-AS1 promotes gastric cancer progression
Source: BMC Cancer. 2023 Mar 20;23:258. doi: 10.1186/s12885-023-10707-7 (PMC10026485; doi:10.1186/s12885-023-10707-7)
Supplement: Supplementary file 4 — Supplementary Material 4 [file 12885_2023_10707_MOESM4_ESM.docx]

Where gels/blots are used in graphics, we ensure their compliance with the digital image and integrity policies.

Our blots were all cut prior to hybridization with antibodies. Raw images of all blots with membrane edges visible and an explanation for the lack of images of sufficient length included where appropriate in the manuscript.（We provide a complete, raw, unprocessed western blot for each antibody at the bottom of this file to confirm specific detection of the target antigen.）

Fig1


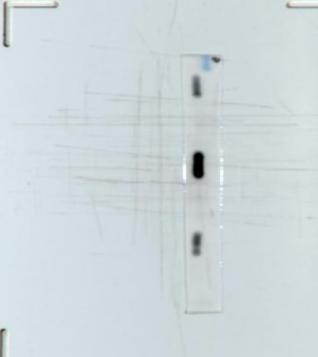

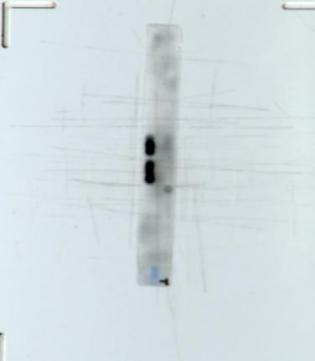
F

GAPDH

36KD

55KD

TUBA1C

The grouping of blots cropped from different parts of the same gel. The blots were cut prior to hybridisation with antibodies.


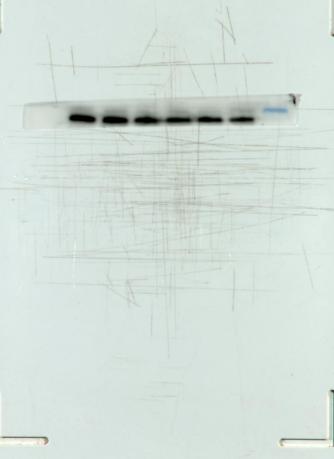


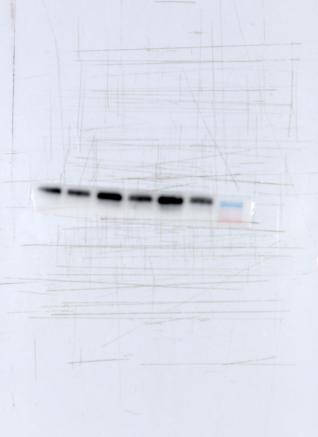


G

MKN-45

group 1 group 2 group 3

55KD

TUBA1C

group 1 group 2 group 3

36KD

GAPDH


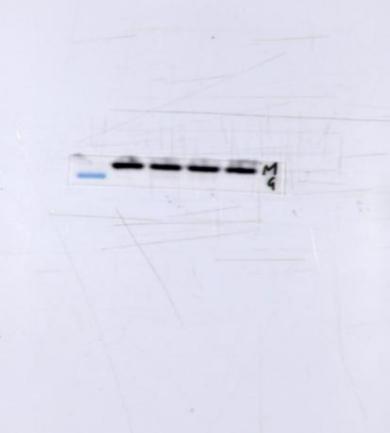

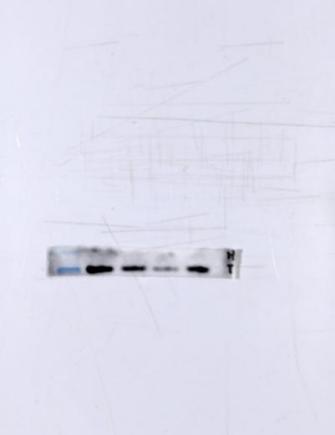


HGC-27

（group1）

36KD

GAPDH

55KD

TUBA1C


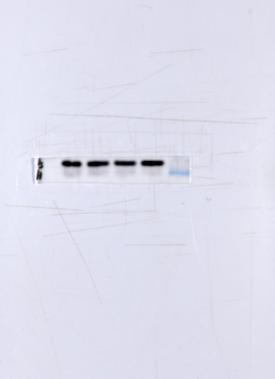

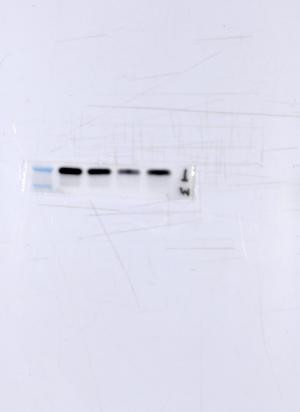


（group2）

36KD

GAPDH

55KD

TUBA1C


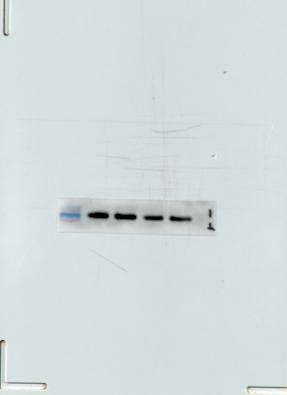

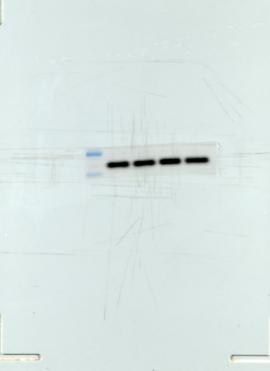


（group3）

36KD

GAPDH

55KD

TUBA1C

The grouping of blots cropped from different parts of the same gel. The blots were cut prior to hybridisation with antibodies.

Fig2


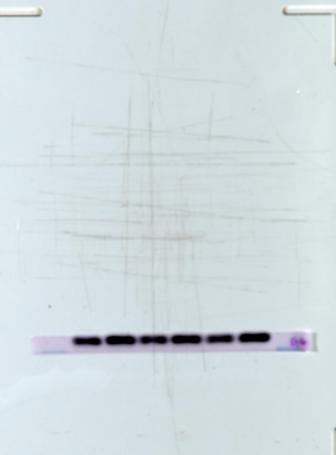

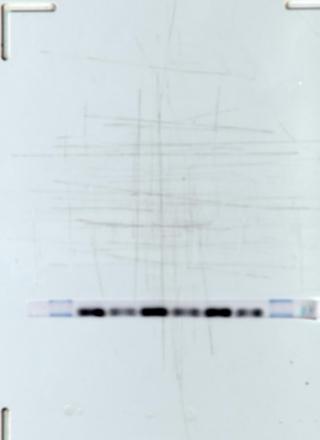
MKN-45

group 1 group 2 group 3

55KD

TUBA1C

GAPDH

group 1 group 2 group 3

36KD

The grouping of blots cropped from different parts of the same gel. The blots were cut prior to hybridisation with antibodies.


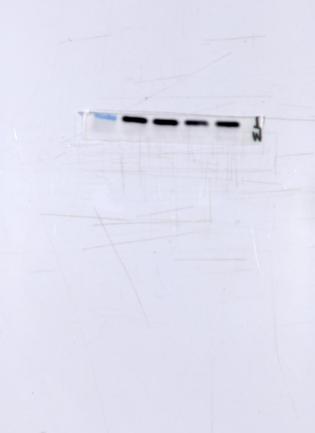


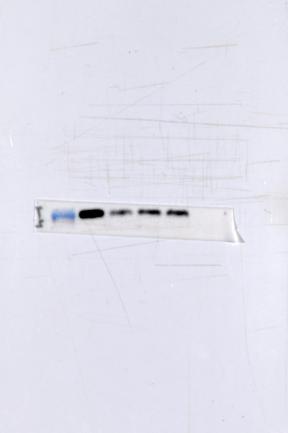
HGC-27

（group1）

55KD

TUBA1C

36KD

GAPDH

（group2）


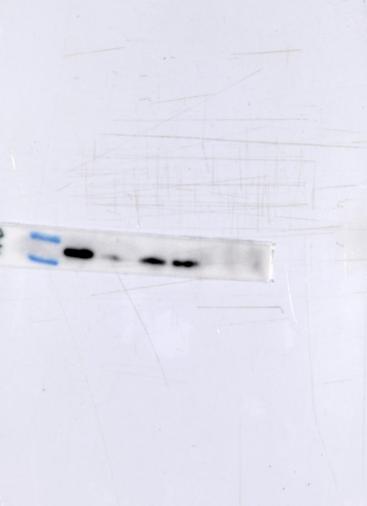

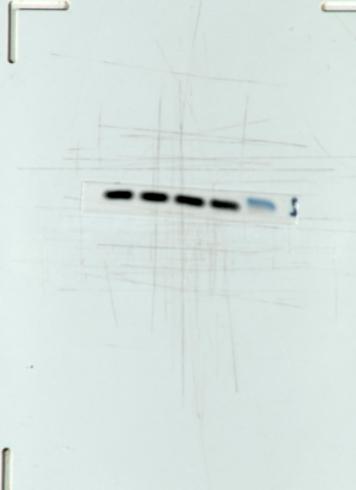


55KD

GAPDH

36KD

TUBA1C

（group3）


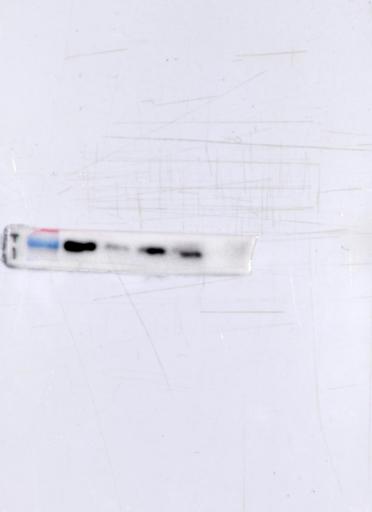


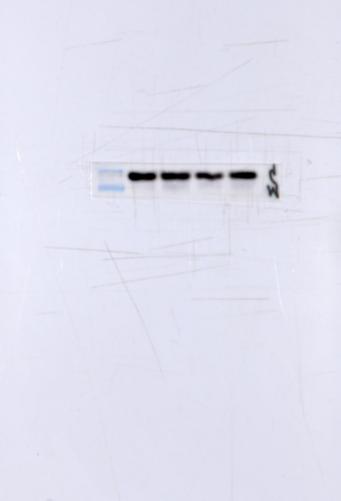


55KD

36KD

GAPDH

TUBA1C

The grouping of blots cropped from different parts of the same gel. The blots were cut prior to hybridisation with antibodies.


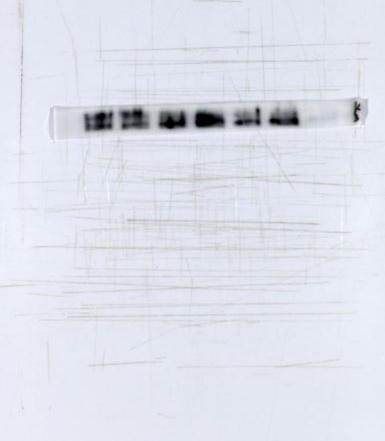

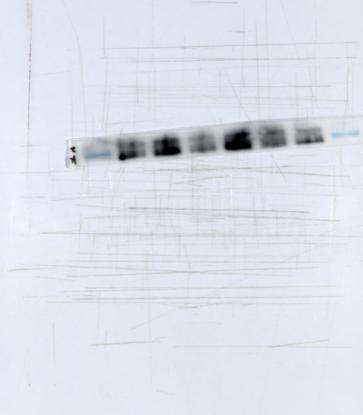
Fig4

MKN-45 HGC-27

group 1 group 2 group 3

KI-67

group 1 group 2 group 3

358KD


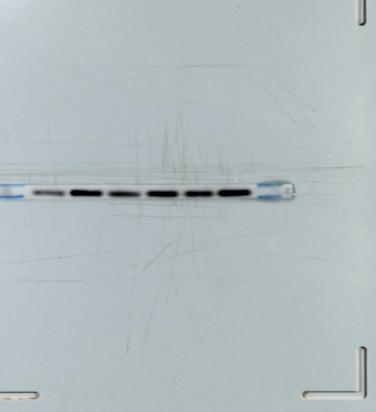

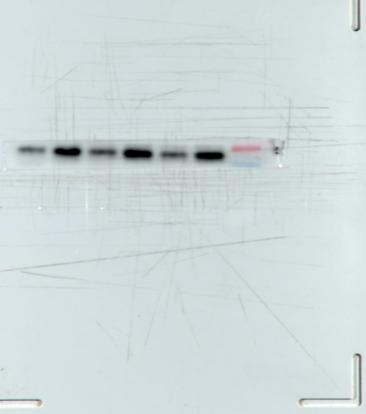


Calculated molecular weight: 47KD

Observed molecular weight：60KD

group 1 group 2 group 3

group 1 group 2 group 3

E2F1


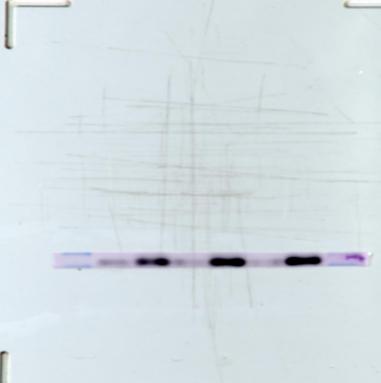

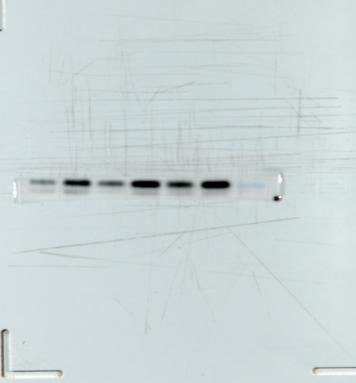


PCNA

group 1 group 2 group 3

36KD

group 1 group 2 group 3


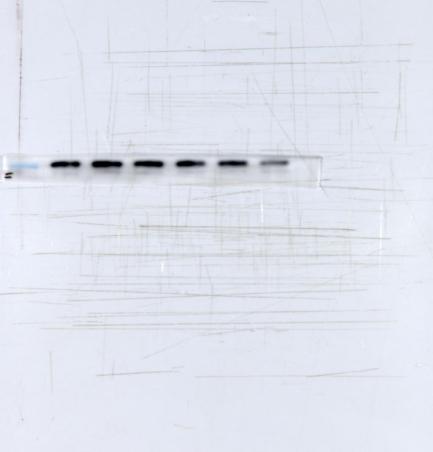


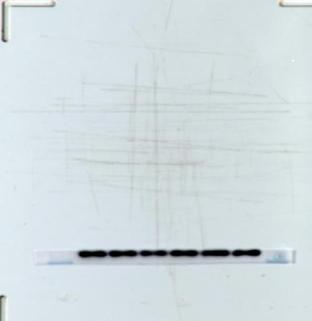


group 1 group 2 group 3

group 1 group 2 group 3

43KD

β-ACTIN

The grouping of blots cropped from different parts of the same gel.The blots were cut prior to hybridisation with antibodies.

MKN-45 HGC-27


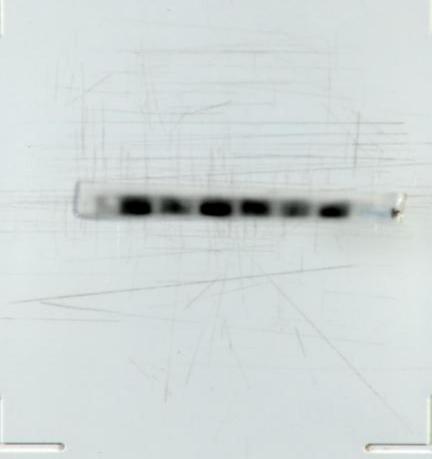
Fig5


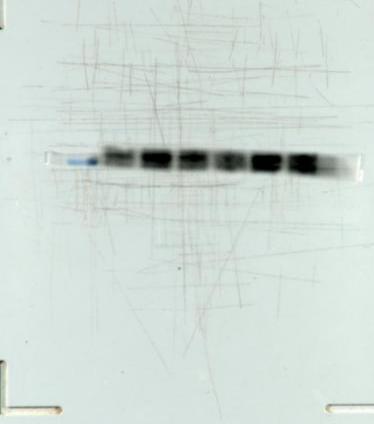


group 1 group 2

358KD

KI-67


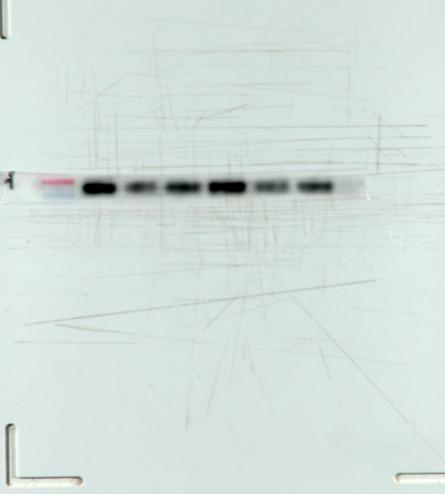


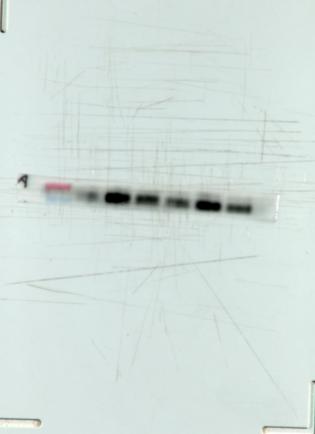


Calculated molecular weight: 47KD

Observed molecular weight：60KD

E2F1

group 1 group 2

group 1 group 2


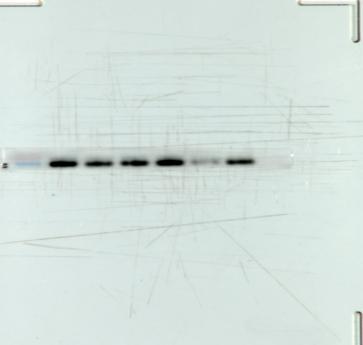

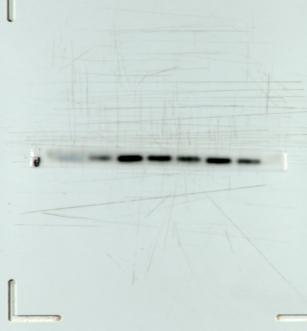


Pcna

group 1 group 2

group 1 group 2

36KD


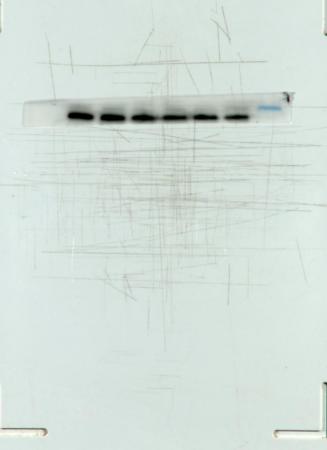


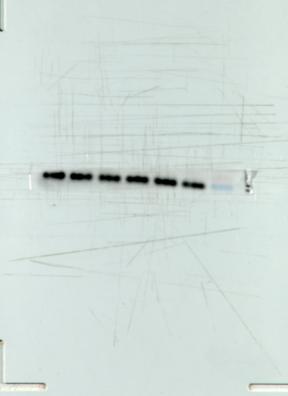


β-ACTIN

group 1 group 2

group 1 group 2

43KD

The grouping of gels/blots cropped from different parts of the same gel.The blots were cut prior to hybridisation with antibodies.


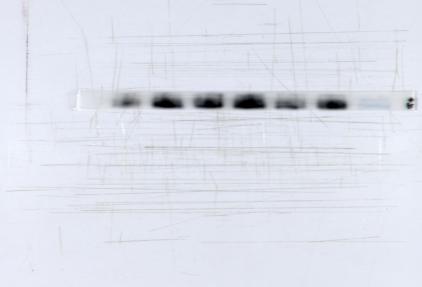


Fig 6

358KD

Ki-67

group 1 group 2 group 3


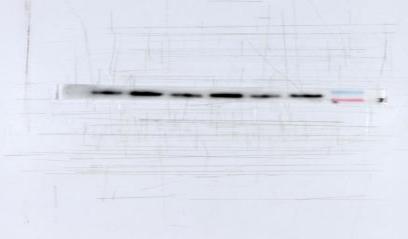


Calculated molecular weight: 47KD

Observed molecular weight：60KD

E2f1

group 1 group 2 group 3


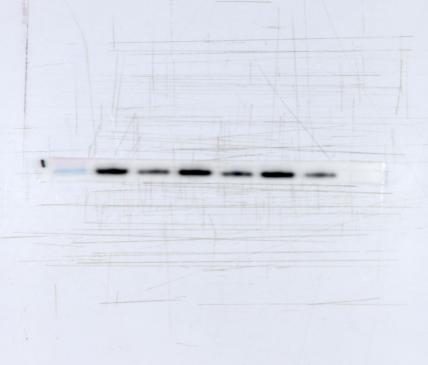


group 1 group 2 group 3

Pcna

36KD


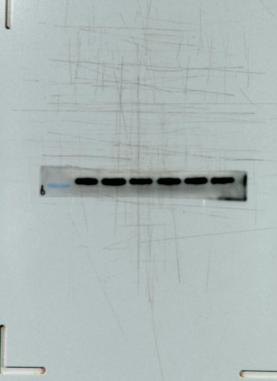


group 1 group 2 group 3

β-ACTIN

43KD

The grouping of blots cropped from different parts of the same gel.The blots were cut prior to hybridisation with antibodies.

Complete membranes

Si-nc si-TUBA1C

Si-nc si-EGFR-AS1


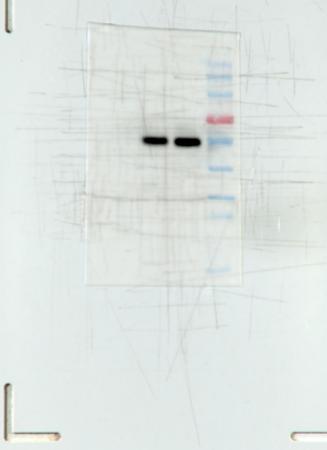

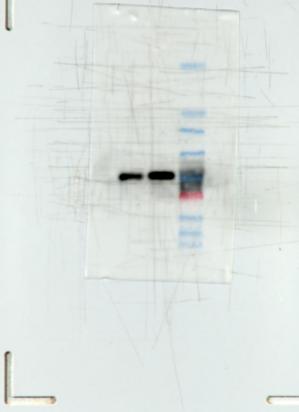


55KD

TUBA1C


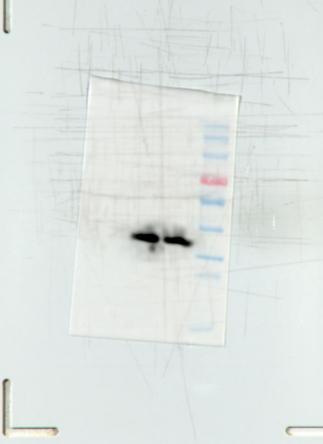

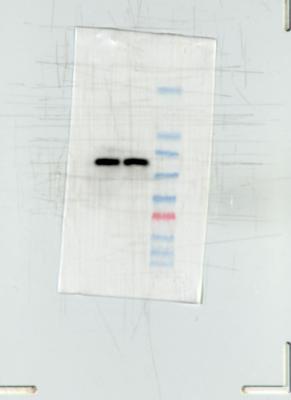


GAPDH

36KD

（Supplementary figure.To verify antibody specificity.)


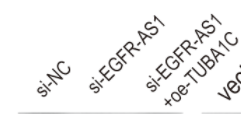

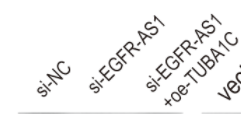

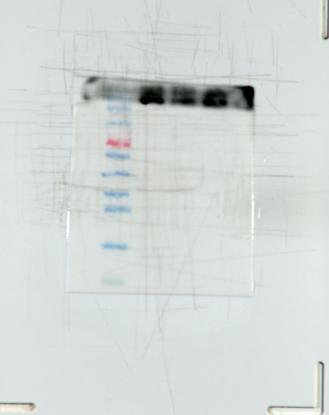


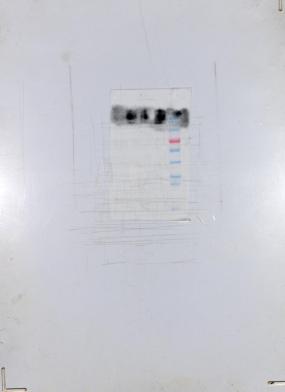


KI-67

358KD


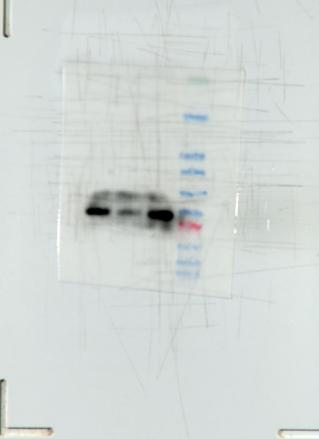


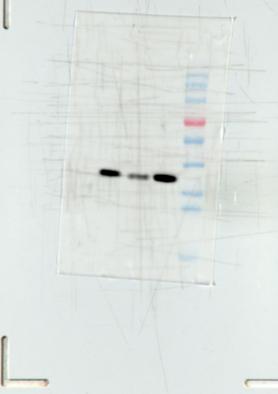


Calculated molecular weight: 47KD

Observed molecular weight：60KD

36KD

PCNA

E2F1


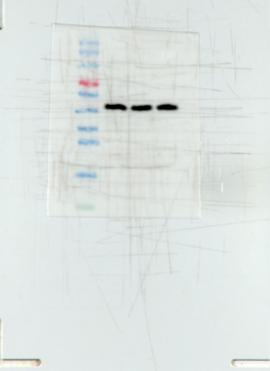

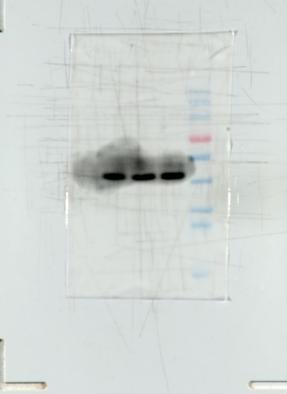


β-ACTIN

43KD
